# Peak width of skeletonized mean diffusivity and cognitive performance in cerebral amyloid angiopathy

**DOI:** 10.3389/fnins.2023.1141007

**Published:** 2023-04-03

**Authors:** Mitchell J. Horn, Elif Gokcal, J. Alex Becker, Alvin S. Das, Kristin Schwab, Maria Clara Zanon Zotin, Joshua N. Goldstein, Jonathan Rosand, Anand Viswanathan, Jonathan R. Polimeni, Marco Duering, Steven M. Greenberg, M. Edip Gurol

**Affiliations:** ^1^Department of Neurology, J. Philip Kistler Stroke Research Center, Massachusetts General Hospital, Boston, MA, United States; ^2^Division of Nuclear Medicine and Molecular Imaging, Department of Radiology, Massachusetts General Hospital, Boston, MA, United States; ^3^Department of Neurology, Beth Israel Deaconess Medical Center, Harvard Medical School, Boston, MA, United States; ^4^Department of Medical Imaging, Hematology and Clinical Oncology, Ribeirão Preto Medical School, Center for Imaging Sciences and Medical Physics, University of São Paulo, Ribeirão Preto, São Paulo, Brazil; ^5^Department of Emergency Medicine, Massachusetts General Hospital, Harvard Medical School, Boston, MA, United States; ^6^Athinoula A. Martinos Center for Biomedical Imaging, Massachusetts General Hospital, Charlestown, MA, United States; ^7^Medical Image Analysis Center (MIAC), Department of Biomedical Engineering, University of Basel, Basel, Switzerland

**Keywords:** cerebral amyloid angiopathy (CAA), cerebral small vessel disease, cognitive functions, diffusion-weighted imaging, peak width of skeletonized mean diffusivity (PSMD)

## Abstract

**Background:**

Cerebral Amyloid Angiopathy (CAA) is a cerebral small vessel disease that can lead to microstructural disruption of white matter (WM), which can be measured by the Peak Width of Skeletonized Mean Diffusivity (PSMD). We hypothesized that PSMD measures would be increased in patients with CAA compared to healthy controls (HC), and increased PSMD is associated with lower cognitive scores in patients with CAA.

**Methods:**

Eighty-one probable CAA patients without cognitive impairment who were diagnosed with Boston criteria and 23 HCs were included. All subjects underwent an advanced brain MRI with high-resolution diffusion-weighted imaging (DWI). PSMD scores were quantified from a probabilistic skeleton of the WM tracts in the mean diffusivity (MD) image using a combination of fractional anisotropy (FA) and the FSL Tract-Based Spatial Statistics (TBSS) algorithm (www.psmd-marker.com). Within CAA cohort, standardized z-scores of processing speed, executive functioning and memory were obtained.

**Results:**

The mean of age and sex were similar between CAA patients (69.6 ± 7.3, 59.3% male) and HCs (70.6 ± 8.5, 56.5% male) (*p* = 0.581 and *p* = 0.814). PSMD was higher in the CAA group [(4.13 ± 0.94) × 10^–4^ mm^2^/s] compared to HCs [(3.28 ± 0.51) × 10^–4^ mm^2^/s] (*p* < 0.001). In a linear regression model corrected for relevant variables, diagnosis of CAA was independently associated with increased PSMD compared to HCs (*ß* = 0.45, 95% CI 0.13–0.76, *p* = 0.006). Within CAA cohort, higher PSMD was associated with lower scores in processing speed (*p* < 0.001), executive functioning (*p* = 0.004), and memory (0.047). Finally, PSMD outperformed all other MRI markers of CAA by explaining most of the variance in models predicting lower scores in each cognitive domain.

**Discussion:**

Peak Width of Skeletonized Mean Diffusivity is increased in CAA, and it is associated with worse cognitive scores supporting the view that disruption of white matter has a significant role in cognitive impairment in CAA. As a robust marker, PSMD can be used in clinical trials or practice.

## Introduction

Cerebral amyloid angiopathy (CAA), which is pathologically characterized by the deposition of amyloid beta (Aβ) peptides in the media and adventitia of small arteries and capillaries of the meninges and cerebral cortex, is a common type of sporadic cerebral small vessel disease (cSVD) and an essential contributor to cognitive impairment in elderly (Gurol and Greenberg, 2018). CAA is known as a significant etiology of lobar intracerebral hemorrhage (ICH), lobar cerebral microbleeds (CMB), and cortical superficial siderosis (cSS) in the elderly (Greenberg et al., 1993; Knudsen et al., 2001; Linn et al., 2010). In addition to these hemorrhagic lesions, CAA is also associated with ischemic brain injury in the form of white matter hyperintensities (WMH), cerebral cortical microinfarcts (CMI), and lobar lacunes (Gurol et al., 2013; Charidimou et al., 2016; Pasi et al., 2017; Xiong et al., 2018; Gokcal et al., 2021). Beyond these imaging markers readily visible on conventional radiological scans, CAA causes microstructural network alterations as quantified using Diffusion Tensor Imaging (DTI). This disruption of brain networks might eventually lead to neurological dysfunction, including cognitive impairment (Salat et al., 2006; Reijmer et al., 2015).

The use of conventional DTI-based metrics has been proposed as a potential biomarker of CAA in assessing disease burden and its clinical consequences (Smith et al., 2019). However, this imaging modality has its limitations, such as the variability in scan parameters and analytic techniques used in analyses and the need for extensive data processing (Gurol et al., 2020). To overcome these weaknesses, another diffusion-based imaging marker, derived using a robust and fully automated method, has been developed (Baykara et al., 2016). The so-called “peak width of skeletonized mean diffusivity” (PSMD), which was derived from a histogram of the mean diffusivity (MD) in skeletonized white matter, strongly correlated with processing speed and consistently outperformed conventional imaging markers of cSVD in different populations such as healthy controls, hereditary and sporadic cSVDs; but not in Alzheimer’s disease (AD) (Baykara et al., 2016).

Previous studies on CAA investigated the association between PSMD and cognitive functions in different cohorts, such as patients with CAA with mild cognitive impairment (MCI), patients from the memory clinic, or patients with lobar CMBs only (McCreary et al., 2020; Raposo et al., 2021; Zotin et al., 2022). The results of these studies demonstrating the associations of PSMD with different cognitive domains might be due to the limited sample size of these studies or the potential confounding effect of Alzheimer’s disease pathology.

In this study, we compared PSMD measures in a cohort of CAA patients with lobar ICH and/or lobar CMBs who did not have cognitive impairment to healthy controls (HC). We investigated the association of PSMD with cognitive domains in patients with CAA. Our hypothesis was that PSMD measures would be increased in CAA patients compared to HCs, and increased PSMD would be associated with lower cognitive performance in different domains in patients with CAA without cognitive impairment.

## Materials and methods

### Study design and participants

Patients were included in an ongoing single-center prospective longitudinal cohort study on the natural history of CAA. Patient selection and inclusion criteria of the study were described elsewhere (Gokcal et al., 2021). Briefly, patients presented with lobar ICH, transient focal neurological symptoms, or other neurological problems except memory decline were diagnosed with probable CAA based on Boston criteria version 1.0 (Knudsen et al., 2001). General cognition was assessed by history taking and brief cognitive tests, including orientation, registration, and recall during the first clinical visit. All patients had preserved general cognitive functions and related functional abilities. Patients who fulfilled the criteria for mild cognitive impairment, dementia, or any neurodegenerative disease based on clinical and imaging evaluations were excluded from the study (McKhann et al., 1984; Petersen, 2004). All participants underwent high-resolution structural MRI at 3T, including diffusion-weighted imaging (DWI). This study also enrolled HCs who did not have any neurological or psychiatric diseases or any type of cognitive complaints. In all participants, hypertension was defined by the ongoing use of an antihypertensive medication or measurement of elevated blood pressure (systolic blood pressure ≥140 mm Hg or diastolic ≥90) on two separate readings in the hospital or outpatient clinic, and hyperlipidemia was defined by documented diagnosis of this disorder in the subject’s medical record.

The study was performed with the approval of and in accordance with the guidelines of the institutional review board of Massachusetts General Hospital and all participants provided written informed consent for the study. There were no photographs, videos, or other information of any recognizable person.

### Structural MRI

All patients and HCs had the same structural MRI sequences obtained in a Siemens (Siemens Healthcare, Erlangen, Germany) Prisma*^fit^* 3T scanner. The protocol included a 3-dimensional (3D) T_1_-weighted multi-echo magnetization-prepared rapid gradient echo (MEMPRAGE: 1 mm × 1 mm × 1 mm voxel size), axial 3D fluid-attenuated inversion recovery (FLAIR) (0.9 mm × 0.9 mm × 0.9 mm voxel size) and high-resolution susceptibility-weighted imaging (SWI: 0.9 mm × 0.9 mm × 1.4 mm voxel size) sequences.

All MRI sequences were reviewed to identify the presence of ICH, the number of lobar CMBs, and the presence and extent of cSS based on established criteria (Greenberg et al., 2009; Charidimou et al., 2015). Lacunes and cortical CMI were also identified using previously defined criteria (Wardlaw et al., 2013; van Veluw et al., 2016; Gokcal et al., 2021). None of the HCs had any hemorrhagic marker of cSVD (ICH, CMBs, or cSS) on MRI. Average cortical thickness (CTh), WMH volume, white matter volume (WMV), and estimated total intracranial volume (eTIV) were calculated using the FreeSurfer software suite (v7.1.1).^1^ WMH and WMV were expressed as a percent of eTIV (pWMH and pWMV, respectively) to correct the measurements for the variable head size of the participants. In patients with ICH, the volume estimates of the ICH-free hemispheres were used and multiplied by 2.

### Diffusion MRI and PSMD analysis

As part of the MRI, all subjects underwent high angular resolution diffusion imaging (HARDI, 2 mm × 2 mm × 2 mm voxel size, *b*-value 700, 1 b0 image, 64 directions). The raw diffusion scans were preprocessed with a state-of-the-art custom pipeline. Images were denoised and removed of Gibbs artifacts using the dwidenoise and mrdegibbs functions of the MRtrix3 package (Tournier et al., 2019). Next, images were corrected for eddy currents and field distortions with the eddy and fugue tools of the Functional Magnetic Resonance Imaging of the Brain (FMRIB) software library (FSL; v6.0) (Woolrich et al., 2009; Andersson and Sotiropoulos, 2016).

Once preprocessed, subjects with WM penetrating ICHs were identified for lesion masking. First, subjects’ high-resolution T1w MEMPRAGEs were imported to the ITK-SNAP toolbox (v3.6.0)^2^ (Yushkevich et al., 2006). Using the ITK-SNAP seed-growing algorithm, volumetric ICHs were segmented and exported in T1w-space. Next, leveraging the cross-modality transform from the FreeSurfer dtrecon process, the lesion was moved into the native DWI space. Lastly, due to the disparity in voxel size across the two imaging modalities, the lesion mask was enlarged using a 3-mm Gaussian blur.

**FIGURE 1:**
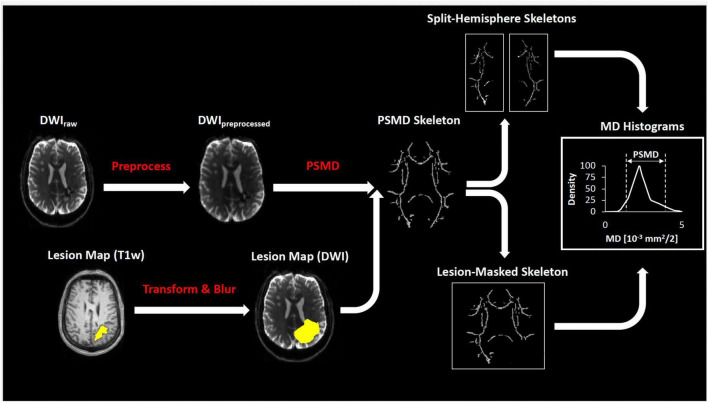
Diagram of diffusion processing. The preprocessed diffusion scans were co-registered with the lesion mask and processed by the fully automated PSMD tool (see section “Materials and methods”) to calculate peak width of the skeletonized MD histogram.

The resulting preprocessed HARDI scans and lesion masks were finally processed by the fully automated PSMD tool (see text footnote 1; v1.8.2). Briefly, the PSMD tool calculates FA and MD from a generic DWI, registers the parametric volumes to a skeletonized WM tract in standardized space, and then calculates the histogram peak width (difference of 95th and 5th percentile) of MD of all voxels within the skeleton (Baykara et al., 2016). Using the newly containerized version of PSMD, subjects were processed in two ways; (Gurol and Greenberg, 2018) whole-brain approach with lesion masks if there was ICH and (Greenberg et al., 1993) separate-hemisphere approach without lesion masks. A diagram of diffusion processing is depicted in Figure 1.

### Cognitive assessment

At study enrollment, patients with CAA were asked to complete a standard neurocognitive test battery. Such data was present in 75 (92.6%) of the CAA cohort. Tests used in separate cognitive domains were as follows: immediate and delayed scores of Hopkins Verbal Learning Test for memory; Trail Making Test A, WAIS-III Digit-Symbol Coding and Digit Span Forward for processing speed; and Trail Making Test B, Digit Span Test backward and Verbal Fluency Test for executive functioning. Each cognitive test score was transformed into a standardized *z*-score based on the CAA cohort’s mean and standard deviation scores. Then *z*-scores of individual tests were averaged into a final score for each cognitive domain (Reijmer et al., 2015).

### Statistical analysis

Both univariable and multivariable models were used to assess (1) the association between PSMD and diagnosis of CAA (vs. HCs) in the whole study cohort, (2) the association of PSMD with demographics, vascular risk factors, and imaging markers within the CAA cohort, and (3) the association between PSMD and z scores of cognitive domains in the CAA cohort with available cognitive data.

In univariable analyses, categorical variables were presented as count (%), and the Pearson chi-square test compared these variables. Continuous variables were presented as mean ± SD or median (IQR), as appropriate, based on their distribution. The independent-sample *t*-test was used to compare normally distributed continuous variables, while the Mann-Whitney *U*-test was used for non-normally distributed variables. Correlation between two continuous variables was assessed using Pearson (for normal distributions) or Spearman (for non-normal distributions) tests.

Separate linear regression models were used for multivariable analyses. The first linear regression model was built to test the independent association between the diagnosis of CAA and PSMD. The second model was set out to assess the independent association of PSMD with demographics, risk factors, and imaging markers of CAA. These models included variables that showed an association with a *p*-value of < 0.1 in univariate analyses as well as those considered to be relevant based on the previous studies, such as age, sex, hypertension, and the presence of ICH.

Lastly, we examined whether PSMD would explain some of the variance in cognitive function on top of other MRI markers by including imaging markers (MD, pWMH, CTh, lobar CMB counts, presence of ICH, lacunes, and microinfarcts) along with age, sex, and years of education into separate linear regression models. We used stepwise linear regression in these models due to the risk of overfitting the model. Finally, we applied a model decomposition method to identify the relative importance of predictors in the regression models. This method is proposed by Lindeman et al. (1980) and implemented in the R package “relaimpo” (version 2.2-3) (Groemping, 2006). pWMH was log-transformed in each regression model due to its non-normal distribution.

In all analyses conducted using the SPSS (Statistical Package for Social Sciences) for Mac Version 26 software or the R statistical software (version 4.0.3), a *p*-value less than 0.05 was considered statistically significant, and all significance tests were 2-tailed.

## Results

Eighty-one patients with probable CAA and 23 were included in the study between January 2015 and August 2020. The baseline characteristics of CAA patients and HCs are presented in Table 1. Neither age nor sex significantly differed between the groups (*p* = 0.581 and *p* = 0.814, respectively).

**TABLE 1:** Baseline characteristics of the study cohort.

	Patients with CAA *n* = 81	Healthy controls *n* = 23	*p*-value
Age, mean ± SD	69.6 ± 7.3	70.6 ± 8.5	0.581
Sex, male *n* (%)	48 (59.3)	12 (56.5)	0.814
Presence of hypertension, *n* (%)	41 (50.6)	0 (0)	–
Presence of hyperlipidemia, *n* (%)	39 (48.1)	0 (0)	–
Presence of lobar ICH, *n* (%)	54 (66.7)	0 (0)	–
Lobar CMBs, median (IQR)	0 (0)	0 (0)	–
Deep CMBs, median (IQR)	0 (0)	0 (0)	–
Presence of cSS, *n* (%)	41 (50.6%)	0 (0)	–
Presence of lacune, *n* (%)	16 (19.7%)	0 (0)	–
Presence of cortical CMI, *n* (%)	18 (22.2%)	0 (0)	–
Processing speed z score, mean ± SD	(−0.18) ± 0.75	–	–
Executive functioning z score, mean ± SD	(−0.13) ± 0.74	–	–
Memory z score, mean ± SD	(−0.002) ± 0.91	–	–

ICH, intracerebral hemorrhage; CMB, cerebral microbleed; cSS, cortical superficial siderosis; CMI, cerebral microinfarct.

Within the CAA cohort, PSMD obtained from the whole brain was significantly correlated with PSMD obtained unilaterally from one hemisphere (*r* = 0.873, *p* < 0.001 for right and *r* = 0.901, *p* < 0.001 for left). Whole brain PSMD also correlated with PSMD from the non-ICH hemisphere when tested only in patients with ICH (*r* = 0.909, *p* < 0.001). Therefore, whole-brain PSMD values -obtained by the lesion masking when ICH was present- were used for the rest of the analyses.

**FIGURE 2:**
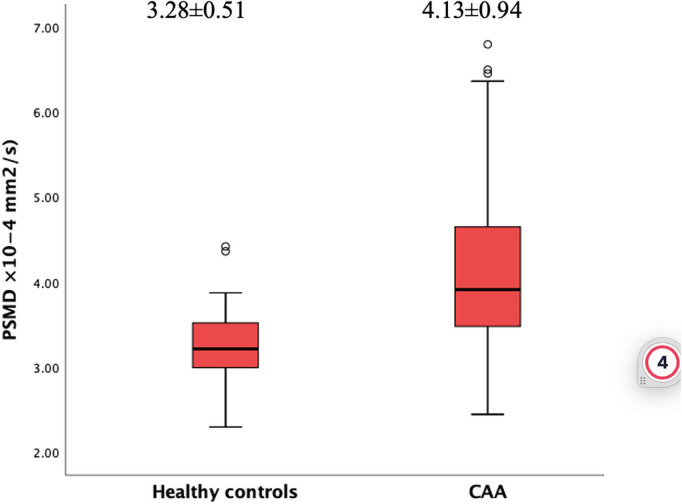
Boxplot of PSMD values in patients with CAA and healthy controls.

When the two groups were compared, patients with CAA had increased PSMD values (4.13 ± 0.94 × 10^–4^ mm^2^/s) than HCs (3.28 ± 0.51 × 10^–4^ mm^2^/s) (*p* < 0.001, Figure 2). This association remained significant in a linear regression analysis corrected for age, sex, hypertension, and hyperlipidemia (*ß* = 0.72, 95% CI 0.27–1.17, *p* < 0.001). In this model, older age and male sex were also independently associated with higher PSMD (*p* = 0.004, *p* = 0.034, respectively).

**FIGURE 3:**
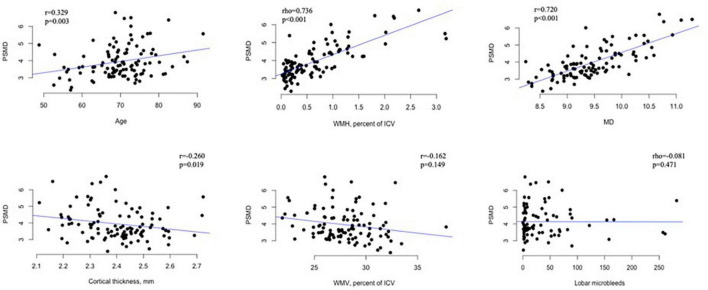
Scatter plots showing the relationship between PSMD (× 10^– 4^ mm^2^/s) and continuous variables in patients with CAA. PSMD, peak skeletonized mean diffusivity; MD, mean diffusivity; WMH, white matter hyperintensity; WMV, white matter volume; ICV, intracranial volume.

Within the CAA cohort, the association of PSMD with demographics, risk factors, and imaging markers of CAA is presented in Table 2 for categorical variables and in Figure 3 for continuous measurements. Increased PSMD significantly correlated with older age (*p* = 0.003), higher WMH (*p* < 0.001), and lower CTh (*p* = 0.019). Male patients had significantly higher PSMD values compared to female patients (*p* = 0.048). PSMD showed a trend to be increased in patients with hypertension than in patients without hypertension (*p* = 0.093) and in patients with hyperlipidemia compared to those without (*p* = 0.066). The PSMD values were not associated with ICH, cSS, lacune, cortical CMI, lobar CMB counts, or pWMV (*p* > 0.1 for all comparisons).

**TABLE 2:** The relationship between peak width of skeletonized mean diffusivity (PSMD) and categorical variables, including demographics, vascular risk factors, and imaging markers in patients with cerebral amyloid angiopathy.

	PSMD	*p*-value
**Sex**		
Male	4.31 ± 0.98	0.048
Female	3.88 ± 0.84	
**Hypertension**		
Yes	4.31 ± 0.88	0.093
No	3.95 ± 0.98	
**Hyperlipidemia**		
Yes	4.33 ± 1.00	0.066
No	3.95 ± 0.85	
**Intracerebral hemorrhage**		
Yes	4.21 ± 0.92	0.309
No	3.98 ± 0.98	
**Cortical superficial siderosis**		
Yes	4.27 ± 0.92	0.181
No	3.99 ± 0.95	
**Lacune**		
Yes	4.01 ± 0.95	0.553
No	4.16 ± 0.94	
**Cortical cerebral microinfarct**		
Yes	4.03 ± 0.81	0.617
No	4.16 ± 0.98	

In a linear regression analysis including age, sex, presence of ICH, hypertension, hyperlipidemia, pWMH, and CTh as independent variables and PSMD as the dependent variable, only higher pWMH (*ß* = 1.40, 95% CI 1.05–1.75, *p* < 0.001) was independently associated with increased PSMD.

**FIGURE 4:**
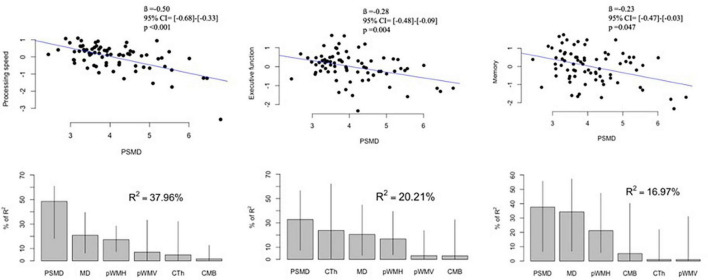
Associations between PSMD and cognitive scores in patients with cerebral amyloid angiopathy: Upper panel shows correlation plots between PSMD (× 10^– 4^ mm^2^/s) and z scores of processing speed, executive function, and memory. The values of ß, 95% CI and p were adjusted for age, sex, and years of education. Lower panel demonstrates the contribution of each MRI marker to the multiple regression models. Lines represent 95% confidence intervals after bootstrapping. PSMD, peak skeletonized mean diffusivity; MD, mean diffusivity; pWMH, percent of white matter hyperintensity to estimated intracranial volume; pWMV, percent of white matter volume to estimated intracranial volume; CTh, cortical thickness; CMB, cerebral microbleed counts.

Demographics and imaging markers of CAA were not different between patients with cognitive data and those without (*p* > 0.2 for all comparisons). Among the patients with CAA who had the cognitive data, higher PSMD values significantly correlated with lower z scores of processing speed (*r* = −0.592, *p* < 0.001), executive function (*r* = −0.374, *p* = 0.001), and memory function (*r* = −0.372, *p* < 0.001). PSMD remained significantly associated with a decrease in scores of processing speed (*p* < 0.001), executive function (*p* = 0.004), and memory (*p* = 0.047) when adjusted for age, sex, and years of education (Figure 4). MD was also independently associated with z scores of processing speed (*ß* = −0.56, 95% CI ([−0.85]–[−0.27]), *p* < 0.001), executive function (*ß* = −0.37, 95% CI ([−0.67]–[−0.07]), *p* < 0.016), and memory (*ß* = −0.40, 95% CI ([−0.75]–[−0.05]), *p* < 0.027).

Stepwise regression models were developed to identify the independent correlates of the z scores in different cognitive domains. These models included MD, pWMH, pWMV, CTh, lobar CMB counts, presence of ICH, cSS, lacunes, and cortical CMIs along with age, sex, and years of education as covariates, and PSMD values were still independently associated with scores of processing speed (*ß* = −0.49, 95% CI ([−0.64]–[−0.33]), *p* < 0.001), executive function (*ß* = −0.30, 95% CI ([−0.48]–[−0.13]), *p* = 0.001), and memory (*ß* = −0.23, 95% CI ([−0.46]–[−0.002]), *p* = 0.048). Eventually, PSMD demonstrated the highest percentage of relative importance among other markers of CAA (MD, pWMH, pWMV, CTh, and lobar CMB counts) in terms of associations with cognitive functions (Figure 4).

## Discussion

In this study, we demonstrated that patients with CAA who do not have MCI/dementia have significant increases in PSMD compared to healthy participants. The increase in PSMD in these CAA patients is independently associated with pWMH and with worse cognitive scores in processing speed, executive function, and memory. Moreover, the association between PSMD and cognitive functions outperformed all other MRI markers of CAA by explaining most of the variance in the models.

Disruption of white matter architecture is a postulated mechanism through which cSVD result in neurological dysfunction. DTI is a sensitive imaging tool to study microstructural network properties that are not visible on conventional MRI by providing indirect measures of the degree of anisotropy and structural orientation of white matter architecture. Previous work using DTI showed impaired structural network connectivity in patients with cSVD and a stronger correlation between cognitive impairment and network disruption than conventional MRI findings (Lawrence et al., 2014; Tuladhar et al., 2016). Studies in patients with CAA have also reported reduced structural network efficiency, particularly in posterior white matter connections, and its association with cognitive dysfunction (Reijmer et al., 2015).

Despite gaining increased popularity in the field of research, DTI has a complex workflow, including extensive data processing and difficulties in the final interpretation (Soares et al., 2013). Therefore, recent years have seen growing interest in using PSMD as a quantitative metric of white matter microstructure. Increased PSMD has been observed in hereditary and sporadic CAA cohorts with limited sample sizes (Schouten et al., 2019; McCreary et al., 2020; Raposo et al., 2021). In one of these studies that included 34 patients with sporadic CAA without dementia, baseline PSMD values were found to be higher in CAA as compared to HCs, mild cognitive impairment (MCI), and AD and that PSMD values were associated with worse scores in processing speed in patients with CAA (McCreary et al., 2020). Another study that compared PSMD between 24 patients with CAA without ICH who also had MCI and 62 patients with MCI not attributable to CAA reported higher PSMD in patients with CAA plus MCI than MCI without CAA and PSMD values in patients CAA plus MCI were associated with worse performance in processing speed (Raposo et al., 2021). A recent study from a memory clinic cohort reported that PSMD was higher in CAA compared to patients without cSVD, and higher PSMD was associated with worse scores in executive functioning and processing speed in the CAA group (Zotin et al., 2022). Our results confirm that the diagnosis of CAA was associated with increased PSMD compared to HCs in a larger patient cohort without clinically evident cognitive impairment. Our cohort included patients with CAA-related ICH and those with lobar CMBs only (about one-third of the cohort). Using ICH presence as a covariate did not change any results obtained in multiple regression models. In addition, we did not include patients with cognitive impairment or dementia at study enrollment, as this is the best way of minimizing the potential confounding effect of Alzheimer’s pathology *in vivo*. This is a very important issue as studies geared toward improving cognitive impairment performed in already demented patients have consistently failed, and the new paradigm is to intervene before clinically overt cognitive dysfunction occurs (Zlokovic et al., 2020). Our study clearly shows that PSMD would be a sensitive marker associated with cognition in a CAA cohort without cognitive impairment.

One of the findings of our study was the association of PSMD with older age. Although the interpretation of this finding is limited due to the cross-sectional design of our study, previous work on healthy participants demonstrated longitudinal changes in DTI metrics other than PSMD during aging (Beaudet et al., 2020). In addition, a recent cross-sectional study evaluating PSMD and other DTI measures among 20,000 participants (aged 19 to 92) from different population-based cohorts reported that only PSMD constantly increased with increasing decades (Sexton et al., 2014).

Our study also confirms the correlation between PSMD and WMH burden, as previously reported in different cohorts (Baykara et al., 2016; Schouten et al., 2019; McCreary et al., 2020; Raposo et al., 2021). This result supports the notion that ischemic brain injury that is mainly reflected by WMH burden contributes to the disruption of white matter architecture and eventually result in increased PSMD. The absence of a relationship between PSMD and other markers of CAA, such as microbleeds, strengthens our view that CAA-related ischemic and hemorrhagic brain injuries might have different pathophysiological mechanisms (Gokcal et al., 2021).

Finally, we found strong associations between higher PSMD and lower scores in processing speed, executive functioning, and memory. Based on correlation coefficients, the strongest association was seen with processing speed and the weakest with memory scores. Moreover, PSMD showed the strongest contribution to low scores of cognitive domains compared with imaging markers of CAA, which was highest for processing speed, followed by executive functioning and memory, as previously reported in a recent study (Durrani et al., 2023). MD was also associated with lower cognitive scores, but PSMD outperformed MD and other markers in the relative importance in multiple regression model for cognitive scores. The first study that developed and validated PSMD in different cohorts of cSVD also demonstrated a strong association between PSMD and processing speed which was the only cognitive domain the study included (Baykara et al., 2016). Previously mentioned studies on CAA analyzed the association of PSMD with scores of executive functioning and memory along with processing speed, but they only found a significant relationship between higher PSMD and lower scores of processing speed (McCreary et al., 2020; Raposo et al., 2021). This might be due to the limited sample size of these studies compared to our sample size. Nevertheless, the stronger association of PSMD with processing speed compared to other cognitive domains in our study supports the idea that processing speed function is more closely related to the global structural integrity of white matter architecture than other cognitive domains (Turken et al., 2008; Penke et al., 2010; Wen et al., 2011). We believe that investigating the relationship between regional connectivity and cognitive domains, i.e., diffusivity alterations in temporal region and memory, would increase our understanding of the anatomical pathways in different cognitive functions in patients with CAA and other cSVD.

Strengths of this study are the well-characterized and large cohort of patients with probable CAA who did not have cognitive impairment, using the same scanner through the study and performance of a comprehensive neurophysiological battery. A limitation is the limited sample size of HCs. We therefore included age and sex to multiple regression models used in the study. It is also clear that the smaller HC cohort could have led to failure to reject the null hypothesis, but we have obtained significant differences. About two-thirds of CAA cohort had lobar ICH that might disrupt the skeletonization of white matter architecture and therefore generating accurate PSMD values. To overcome this issue, we masked ICH and peri-ICH gliosis in patients with ICH. In addition, the PSMD skeleton generated in each patient with ICH was visually inspected and the rim of the masked area was increased when necessary to avoid any potential effect from ICH-related parenchymal injury. Finally, the presence of ICH was included as a covariate in every multiple regression model built in the study.

In conclusion, CAA even in the absence of cognitive impairment is associated with global microstructural alteration compared to HCs, as demonstrated by increased PSMD and this alteration is related to poorer cognitive performance in patients with CAA. As a robust marker, PSMD might be used in clinical practice or therapeutic trials.

## Data availability statement

The raw data supporting the conclusions of this article will be made available by the authors, without undue reservation.

## Ethics statement

The studies involving human participants were reviewed and approved by the Institutional Review Board of Massachusetts General Hospital. The patients/participants provided their written informed consent to participate in this study.

## Author contributions

MH: literature search, study design, data collection, imaging data analysis, statistical analysis, and manuscript writing. EG: literature search, study design, data collection, statistical analysis, and manuscript writing. MG: study design, data collection, manuscript writing, and critical review of the study. JB, AD, SG, and MG: data collection, analysis of data, and critical review of the study. MZ, JG, KS, JR, JP, and AV: data collection and critical review of the study. All authors contributed to the article and approved the submitted version.

## Footnotes

1
surfer.nmr.mgh.harvard.edu

2
www.itksnap.org
